# Preparation of 12-Tungstophosphoric Acid Embedded in a Silica Matrix and Its Effect on the Activity of 1-Propanol Dehydration

**DOI:** 10.1021/acsomega.4c10379

**Published:** 2025-04-14

**Authors:** Eduardo de Souza Mello Falcão, Deborah da Silva Valadares, Giovana Magalhães dos Santos, Estelle Silva Diorato Teixeira de Mendonça, Marcello Moreira Santos, Sílvia Cláudia Loureiro Dias, José Alves Dias

**Affiliations:** † Universidade de Brasília, Instituto de Química, Laboratório de Catálise, Campus Universitário Darcy Ribeiro, Asa Norte, Brasília, DF 70910-900, Brazil; ‡ Universidade Federal da Bahia, Instituto de Química, Campus Universitário de Ondina, Rua Barão de Jeremoabo, 147, Salvador, BA 40170-115, Brazil; § Universidade de Brasília, Instituto de Química, Laboratório de Degradação e Estabilização de Compostos, Campus Universitário Darcy Ribeiro, Asa Norte, Brasília, DF 70910-900, Brazil

## Abstract

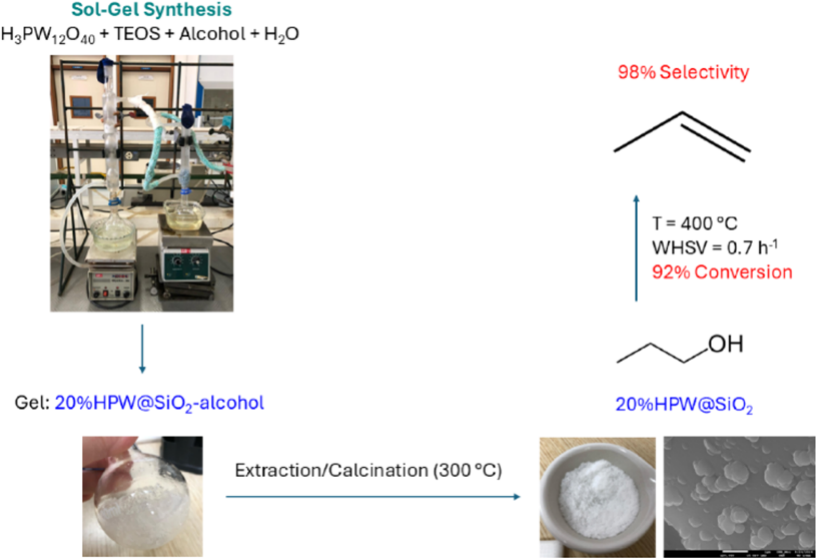

Heteropolyacids (HPAs) such as 12-tungstophosphoric acid (HPW) encapsulated in silica and other matrices are widely used in heterogeneous catalysis because of their enhanced catalytic stability in liquid and gas-phase reactions. In this work, we systematically studied, for the first time, HPW embedded in a silica matrix using different alcohols (methanol, ethanol, 1-propanol, 1-butanol, and 1-octanol) during the sol–gel preparation method. The catalysts, *x*%HPW@SiO_2_-alcohol (*x* = 10, 20, 30, and 40 wt %) were characterized by several methods: energy dispersive X-ray fluorescence (EDXRF), Fourier transform infrared (FT-IR), X-ray diffraction (XRD), ^31^P MAS NMR, N_2_ sorption at low temperature (−196 °C), and scanning electron microscopy/energy dispersive X-ray spectroscopy (SEM/EDS). The HPW Keggin structure was maintained under all preparations. The elemental and leaching tests showed that 20%HPW@SiO_2_-butanol was one of the most promising materials for catalysis. It was found a direct correlation between the textural properties (e.g., Brunauer–Emmett–Teller (BET) specific surface area) and the size of the carbon chain used in the synthesis. The larger the alcohol chain, the greater the tendency to form mesopores, which in turn increase the stability of the entrapped HPW. SEM/EDS and XRD results indicated a good dispersion of HPW on the inner surface of silica. A high conversion of 1-propanol (92%) and selectivity to propene (98%) were obtained with the 20%HPW@SiO_2_-butanol catalyst at 400 °C, which has Brønsted acid sites and a density of around 0.20 H^+^ nm^–2^. These combined properties (BET surface area, mesoporous volume, Brønsted acid density, and dispersion) contributed to enhancing the activity of the catalyst for this reaction under our experimental conditions.

## 1 Introduction

Solid acid catalysts, including heteropolyacids (HPAs), play a crucial role in industrial applications due to their unique catalytic properties.^1−6^ Among HPAs, 12-tungstophosphoric acid (HPW) is extensively employed in both homogeneous and heterogeneous catalysis.^7−9^ HPW is valued for its exceptionally high Brønsted acidity, excellent thermal stability, and its ability to minimize side reactions, particularly at temperatures below 400 °C.^10−13^ Nevertheless, a significant limitation of HPW and other HPAs is their limited specific surface area, which restricts their catalytic performance in surface-dependent reactions. Moreover, its high solubility in water and polar solvents poses challenges for its application in heterogeneous catalysis, leading to efforts aimed at modifying its properties to enhance stability.^14−22^

Current research efforts have focused on encapsulating HPW in solid supports like silica to enhance its surface area, reduce leaching, and improve its overall performance. Leaching, which occurs when the active phase of the catalyst dissolves into the reaction medium, is a frequent issue in supported catalysts, including HPW, and often results in catalyst deactivation.^23,24^ Therefore, it is crucial to develop encapsulated HPW catalysts that maintain high activity and selectivity while being reusable and resistant to leaching.^25−27^

Extensive research has investigated the encapsulation of HPW in silica through the sol–gel method.^28−43^ Pioneering studies conducted by Izumi et al.^28−31^ focused on incorporating HPAs using a sol–gel process with ethanol or n-butanol as solvents and tetraethyl orthosilicate (TEOS) as the silica precursor. This approach yielded silica-included HPA materials with high surface areas and mesoporous structures, significantly enhancing their catalytic properties across various reactions, including esterification, alkylation, and hydrolysis.

Molnár et al.^32^ expanded on these studies by immobilizing various HPAs (e.g., H*_n_*XM_12_O_40_, where *n* = 3 or 4; X = P or Si; M = Mo or W) into silica using a similar sol–gel approach. These composites were applied to the hydrolysis of ethyl acetate, Friedel–Crafts alkylation, and the synthesis of methyl *tert*-butyl ether (MTBE), revealing that silica-entrapped HPAs exhibited enhanced catalytic activity. Kukovecz et al.^33^ further examined HPW-silica composites, particularly for their application in the Diels–Alder reaction between 1,3-cyclohexadiene and 2-propenal. By incorporating 5 to 20 wt % HPW into a TEOS solution, they developed composite catalysts that matched the activity of silica–alumina catalysts. Importantly, these composites demonstrated improved selectivity, which was attributed to the destruction of strong Brønsted acid sites, responsible for side reactions, during the calcination of the dry gel.

Other studies by Farhadi,^34^ Ballarini,^35^ Caetano,^36^ Pito et al.,^37^ and Popa et al.^38^ explored varying HPA loadings in silica composites (ranging from 4 to 74%). These catalysts were tested in diverse processes, such as the photocatalytic oxidation of alcohols, the oxidation of isobutane to methacrolein, the esterification of palmitic acid with methanol, and the methoxylation of α-pinene. The results indicated that silica-entrapped HPA catalysts were efficient, environmentally friendly, and exhibited notable catalytic activity and reusability, while maintaining the Keggin structure.

Other researchers have incorporated surfactants into experimental HPA-silica composite syntheses.^39−43^ For example, Yan et al.^39^ utilized a nonionic surfactant (Brij30) to encapsulate 20 wt % HSiW (silicotungstic acid) into silica. This catalyst demonstrated excellent performance in producing methyl levulinate (ML) and ethyl levulinate (EL), achieving yields of 67% for ML and 75% for EL. Li et al.^40^ employed Pluronic-P123 as a surfactant to encapsulate vanadium-substituted HPW (PMoV_2_) in silica, testing its efficacy in the hydroxylation of benzene to phenol. Similarly, Lv et al.^41^ investigated the transformation of fructose into 5-hydroxymethylfurfural (HMF) using HPW and HSiW encapsulated into silica. Their results highlighted that the modified HSiW catalyst, characterized by its microporous structure and high Brønsted acidity, was highly effective in this reaction. According to these studies, the catalysts were stable, reusable, and suitable for potential industrial applications.

Encapsulation of HPAs into alternative matrices has also been explored in more recent research.^44−54^ These studies aim to broaden the range of materials that enhance HPA performance as heterogeneous catalysts, optimizing active site accessibility, improving reusability, and mitigating leaching.

Propanol dehydration is a critical industrial reaction for producing propene (propylene), especially using renewable sources like propanol. Propene serves as a fundamental building block for a wide range of chemicals, making the development of efficient and sustainable production methods vital for reducing energy consumption and carbon emissions in the chemical industry.^55^ For instance, Motte et al.^56^ recently proposed a renewable 1-propanol production method using a process called C123. Considering the importance of minimizing carbon emissions, more energy-efficient processes for the dehydration of propanol to selectively produce propylene under milder conditions are increasingly sought. In this context, HPAs have been tested for 1-propanol and isopropanol transformation to propene.^57,58^

In this report, we systematically explored the encapsulation of HPW in silica using the sol–gel method, with a focus on evaluating the impact of different alcohols (methanol, ethanol, 1-propanol, 1-butanol, and 1-octanol) on the specific surface area and porosity of the resulting materials. Silica, a low-cost and highly tunable material, offers exceptional surface area and porosity, making it an ideal support for HPW. The acidity and catalytic activity of these composites were assessed in the dehydration of 1-propanol to propene, an important reaction for producing propene from renewable sources.

## 2 Experimental Section

### 2.1 Materials

Commercial solid hydrated 12-tungstosphosphoric acid (H_3_[PW_12_O_40_]·*n*H_2_O, HPW) (99.9%, Sigma-Aldrich) was calcined at 200 °C (muffle furnace, EDG model Seven, Brazil) to form the hexahydrate (H_3_[PW_12_O_40_]·6H_2_O), as determined by TG/DTG analysis, before the utilization on the synthesis of the catalysts. Methanol (p.a., 99.5% CH_3_OH, Synth, Brazil), ethanol (p.a., 99.5% C_2_H_5_OH, Vetec, Brazil), 1-propanol (p.a., 99.5% C_3_H_5_OH, Vetec, Brazil), 1-butanol (p.a., 99.5% C_4_H_9_OH, Neon, Brazil), 1-octanol (p.a., 99.5% C_8_H_17_OH, Neon, Brazil), Tetraethyl orthosilicate (r.g., 98% Si(C_2_H_5_O)_4_, Sigma-Aldrich) and HCl (p.a., 37%, Merck, Brazil) were used without further purification. Deionized water (DI) was produced using a Milli-Q model direct 8, Merck Millipore.

### 2.2 Preparation of HPW Encapsulated in Silica (HPW@SiO_2_)

The quantity of HPW 6H_2_O (1.37; 2.74; 4.11 and 5.48 g) weighed was equivalent to producing a composite with approximately 10, 20, 30, and 40 wt % HPW loading in relation to silica (i.e., HPW/HPW + SiO_2_). The actual value was calculated further by energy dispersive X-ray fluorescence (EDXRF, Shimadzu, model EDX-720, Japan). The reported samples are labeled in the text as nominal loading.

**Scheme 1:**
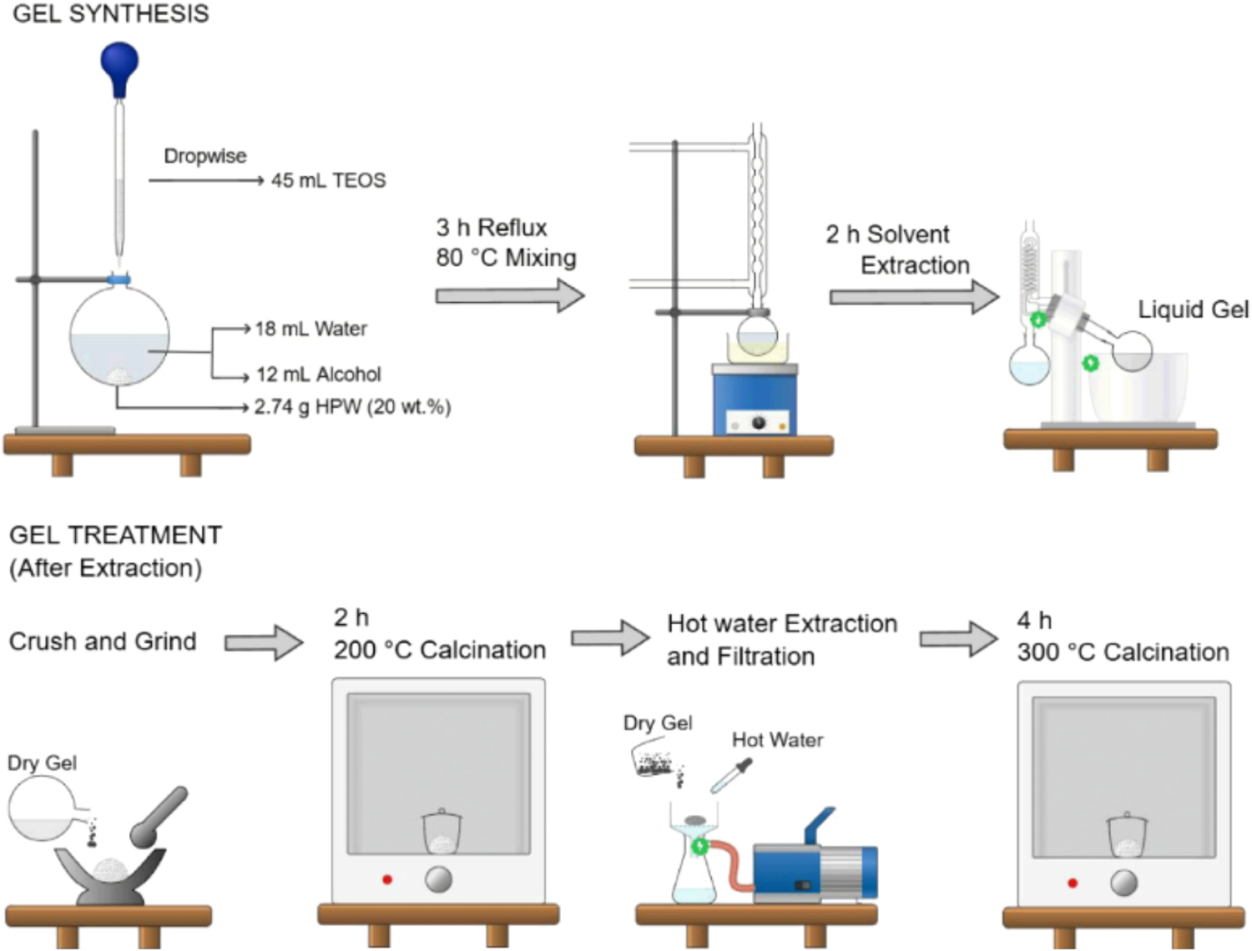
General Diagram of 20%HPW@SiO_2_-Alcohol Synthesis by the Sol–Gel Method

The experimental procedure (Scheme 1) is detailed described here as an example. It is based on the proposed method by Izumi,^30^ but with some modifications. Using a 100 mL round-bottom flask, HPW 6H_2_O (2.74 g, equivalent to 20 wt %) was mixed with 9 mL of deionized water (1 mol) and around 6 mL (0.08 mol) of the following alcohols: methanol, ethanol, 1-propanol, 1-butanol, and later 1-octanol (denoted as MeOH; EtOH; PrOH; BuOH; and OcOH, respectively). The mixture was magnetically stirred, and 23 mL of TEOS (0.1 mol) were added dropwise. The mixture was maintained under constant stirring (500 rpm) while the system was heated to 80 °C and kept under reflux for 3 h. This time was sufficient for the formation of a liquid gel, which was subsequently subjected to extraction at 80 °C for around 2 h in a rotary evaporator (Büchi, RE-120, Switzerland), under vacuum (about 1 × 10^–4^ Torr). After evaporation of the solvent, the mixture was weighed, ground in the mortar, and placed in a muffle furnace at 200 °C for 2 h. After 2 h, the product was weighed again (to check the mass loss of water), followed by extraction with hot water (about 50 mL) to eventually remove any HPW that could be loosely bound to the silica gel. Finally, vacuum filtration was carried out, washing the product with 10 mL of water and the product was calcined for 4 h at 300 °C (to strengthen the bonds of the HPW encapsulated into silica). The obtained product (*x*%HPW@SiO_2_) was quantitatively yielded (around 8 g) because all tetraethyl orthosilicate was completely hydrolyzed. The volumes of TEOS and the alcohol, respectively, were adapted for each synthesis to maintain approximately the same mol ratio between them (i.e., 0.1:0.08). These syntheses were repeated at least 3 times to confirm the reproducibility, as well as being prepared with double the mass of the reactants. In all cases, the yield was about 95% (measured after drying at 200 °C). The yields agree with the literature.^28−31^ The materials were labeled as *x*%HPW@SiO_2_-alcohol, where the alcohol is abbreviated as shown above. The purpose of this alcohol notation is to only indicate the original solvent used in the synthesis.

For comparative purposes, the traditional incipient wetness impregnation method was used for the supported 20 wt % HPW on silica (99.8% SiO_2_, Aerosil 200, Evonik, Germany). The material was designated as 20%HPW/SiO_2_. The detailed preparation method and characterization of this material have been reported elsewhere.^59^

### 2.3 Catalyst Characterizations

The *x*%HPW@SiO_2_ materials have been characterized using different structural, textural, morphologic and acidity methods. Powder diffraction patterns were obtained with a powder diffractometer manufactured by Bruker (model D8 Focus, θ-2θ, Germany) using Cu Kα = 0.15418 nm tube, operating at a power of 40 kV and 30 mA. A scanning rate of 1° min^–1^ at increments of 0.05° was used for the angles ranging from 20–70°.

Fourier transform infrared (FT-IR) spectra were collected using a spectrometer manufactured by Thermo Fisher Scientific (Nicolet, model 6700, MCT detector) in transmittance mode with KBr pellets. The settings used were 4 cm^–1^ resolution and 128 scans for each spectrum.

The textural properties of the materials were obtained by gaseous N_2_ physisorption (including adsorption and desorption branches) at a low temperature (−196 °C) using a surface analyzer manufactured by Micromeritics (model ASAP 2020C). About 0.5 g of the solid was degassed for 6 h at 200 °C under high vacuum (to a target of 20 μmHg) before the physisorption experiment. The data obtained were the specific surface area (BET, at ranges of *P*/*P*_0_ = 0.01–0.20); micropore area (*S*_micro_, t-plot); mesoporous area (*S*_meso_, BJH) and external area (*S*_ext_, t-plot) calculated from the desorption isotherms. The total pore volume (*P*_v_) was obtained at *P*/*P*_0_ = 0.98.

Magic angle spinning solid-state nuclear magnetic resonance (MAS NMR) spectra were obtained using a spectrometer manufactured by Bruker (model Avance III HD, Ascend, Germany), which operates at 14.1 T, 600 MHz for ^1^H and 243.1 MHz for ^31^P). A 4 mm CP/MAS probe and zirconia rotors were used to pack samples. The experimental acquisition conditions for the ^31^P nucleus included: 4.75 μs pulse duration (90°), 10 kHz spin rate; single pulse with 10 s interval and ^1^H decoupling; 512 probing pulses, and utilization of an external secondary reference of ammonium dihydrogen phosphate, NH_4_H_2_PO_4_ (δ = 0.9 ppm), digitally corrected to the primary standard, 85% phosphoric acid, H_3_PO_4_ (δ = 0 ppm).

Microscopy images of the catalysts were obtained using a scanning electron microscope from JEOL (model JSM 7100F, Japan, equipped with a secondary electron detector, LED—low energy detector, under high vacuum at a voltage of 15 kV and magnifications ranging from 100 to 20,000. The measurements also included semiquantitative analysis by energy dispersive X-ray spectroscopy (EDS). The samples were analyzed using a sample holder with carbon tape and coated with carbon.

Catalyst leaching was tested using a method previously developed in our laboratory.^22^ The basic procedure involved adding 50 mL of alcohol (methanol, ethanol, 1-propanol, and 1-butanol) to approximately 0.02 g of the solid catalyst (calcined at 300 °C) at room temperature (25 °C). The slurry was kept under magnetic stirring for 1 h, and every 10 min the stirring was stopped to remove an aliquot using a 1.0 mL gastight syringe (Hamilton) fitted with a membrane filter (0.45 μm, 13 mm diameter). Spectrophotometric analysis was performed using a spectrophotometer from Beckman (model DU 650 UV–vis, USA) with measurements taken at 263 nm (maximum of the [PW_12_O_40_]^3–^ absorption band). The analytical equations obtained for each solution of HPW in the respective solvent are available in the Supporting Information.

### 2.4 Catalytic Reaction of 1-Propanol Dehydration

The catalytic tests for the dehydration of propanol were run in a pulse microreactor coupled to a gas chromatography provided by Shimadzu (model 2010, FID detector, Japan) equipped with a Shimadzu CBP1 PONA-M50–042 column (50.0 m × 0.15 mm × 0.33 μm). Each pulse consisted of an injection of 1-propanol (0.1 μL) into the GC-liner (reactor) containing 10 mg of the catalyst. The programming conditions were: pressure = 95.6 kPa; total flow = 6 mL min^–1^; column temperature = 35 °C; column flow = 0.1 mL min^–1^; linear velocity = 6.4 cm s^–1^; purge flow = 1 mL min^–1^; split ratio = 49. Helium was the carrier gas, and the flame (FID) temperature = 250 °C. These parameters were determined to reach separation of the main product’s peaks, which were about 35 min. The WHSV was calculated by assuming: the mass feed as 0.0803 mg (0.1 μL) of 1-propanol; contact time calculated from helium gas flow and the volume of 1-propanol in gas phase (ideal gas) at 300 °C, reaching mass flow rate of 1.14 × 10^–4^ g min^–1^; and finally, 10 mg of catalyst. Thus, the approximate WHSV was 0.7 h^–1^. The temperature of the reactor was set at: 300, 350, and 400 °C.

The calculations for the conversion of 1-propanol, and selectivity for propene (PP) and dipropyl ether (DPE) were defined by eqs 1 and 2), where *n* is the number of moles of the reactant
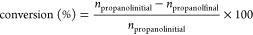
1
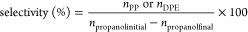
2

## 3 Results and Discussion

### 3.1 Elemental Analyses

#### 3.1.1 Energy Dispersive X-ray Fluorescence (EDXRF)

The loading of HPW was calculated based on W analysis determined by energy dispersive X-ray fluorescence software (Shimadzu), which used the QualiQuant method of fundamental standards. The theoretical values were close to the actual ones, with general deviations of less than 3% (Table 1). Thus, for simplicity, the nominal values were used in this report. We are positive that the main contribution to such an agreement was attributed to the previous thermal treatment of hydrated HPW to form HPW 6H_2_O, which was more stable for weighing. It should also be noted that samples with octanol were only prepared for 20 wt.%, since this alcohol was only screened after the textural and activity results of the materials (see later discussion in the text, Sections 3.3 and 3.6). Thus, the actual loading for the catalyst 20%HPW@SiO_2_–OcOH was 20.3%.

**Table 1:** Composition of the *x*%HPW@SiO_2_-Alcohol Prepared Catalysts after Calcination

nominal/actual HPW@SiO_2_ loading (%)				
catalyst/solvent	10% HPW	20% HPW	30% HPW	40% HPW
methanol	9.9	20.8	31.5	40.1
ethanol	10.2	20.1	30.1	39.8
1-propanol	10.0	19.6	30.3	40.6
1-butanol	10.1	20.4	30.2	40.7

#### 3.1.2 Simulated Leaching Experiments

An important issue related to supported catalysts is leaching. In this case, we conducted tests with the original alcohol used in the synthesis during a 1 h extraction. The solution was sampled every 10 min to verify the presence of Keggin HPW using ultraviolet–visible (UV–vis) spectroscopy for detection in a method already applied for different supported HPW.^22,60−62^ The average leaching for each catalyst (x%HPW@SiO_2_-alcohol) was calculated by the absorbance of the solution, and the results are shown in Table 2. These measurements clearly showed that the catalyst prepared in 1-butanol was the most efficient. In addition, the leaching is generally smaller for lower loadings, and 20%HPW@SiO_2_–BuOH generally had the lowest leaching in the series. Therefore, considering the obtained leaching data, the material of 20%HPW@SiO_2_–BuOH appears to be the most promising for testing in catalytic reactions using polar solvents or probe molecules. In addition, this loading value is frequently found in the literature on supported or encapsulated HPW systems.^27,33,39,59^ In view of these preliminary results, the characterization of the materials with various loadings is provided in the Supporting Information for the sake of conciseness.

**Table 2:** Average Leaching of HPW in Catalysts, Sampled Every 10 min Interval during 1-h Measurement^a^

average leaching *x*%HPW@SiO_2_-ROH (%)				
catalyst/solvent	10% HPW	20% HPW	30% HPW	40% HPW
methanol	1.2	1.1	1.8	2.1
ethanol	0.8	0.9	0.9	1.2
1-propanol	0.5	1.0	1.1	1.4
1-butanol	0.2	0.1	0.3	0.6

a The error is about 0.3%

### 3.2 Structural Analyses

#### 3.2.1 Fourier Transform Infrared Spectroscopy (FT-IR)

**Figure 1:**
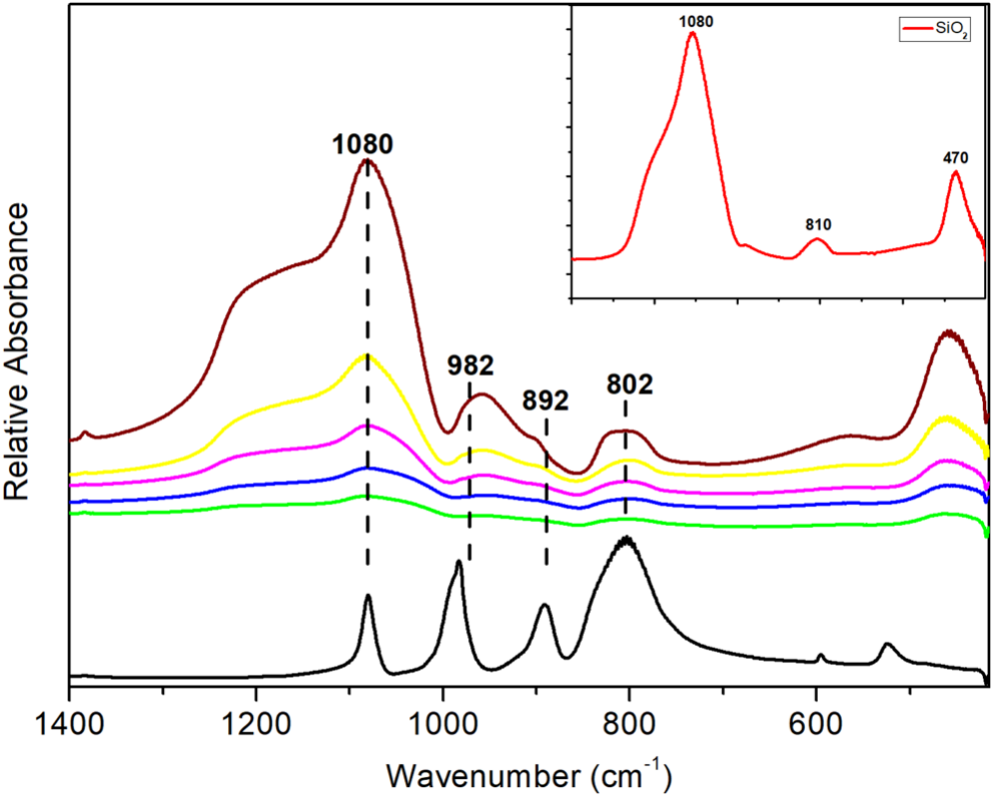
FT-IR spectra of HPW (black) and 20%HPW@SiO_2_-alcohol: MeOH (green); EtOH (blue); PrOH (violet); BuOH (yellow); OcOH (brown). The inset shows the SiO_2_ spectrum.

The FT-IR spectra of the *x*%HPW@SiO_2_-alcohol catalysts were very similar, regardless of the type of alcohol used in the synthesis. Figure S1 shows examples of spectra for 10 to 40% HPW@SiO_2_–BuOH. The loading of 20% HPW using the alcohols is shown in Figure 1 as typical spectra. The original Keggin bands appear to be preserved on the encapsulated samples, but they were broadened and partly obscured because of the strong absorptions of silica gel as observed for supported HPW on silica.^59^ The HPW structure exhibits absorption bands at 1080, 982, 892, and 802 cm^–1^, which are the fingerprint vibrations that represent the specific oxygen atom positions (internal, terminal, corner and edge shared) in the Keggin structure. Silica gel has the main characteristic bands at 1080, 810, and 470 cm^–1^. Thus, the absorption bands of all 20%HPW@SiO_2_-alcohol composites only suggest the presence of the Keggin structure in the material. The bands at 982 and 892 cm^–1^ are slightly visible in almost all materials, with lower visibility in the 20%HPW@SiO_2_-MeOH material and higher visibility in 20%HPW@SiO_2_–OcOH (Figure 1). It was also possible to note that in the encapsulated HPW materials, wider bands were generated than in the supported ones, as evidenced in the band corresponding to 983 cm^–1^ for 20%HPW@SiO_2_–OcOH versus 20%HPW/SiO_2_ (Figure S2). Therefore, to confirm the maintenance of the Keggin structure encapsulated into SiO_2_, it is essential to combine FT-IR with other characterization techniques.

#### 3.2.2 Powder X-ray Diffraction (XRD)

**Figure 2:**
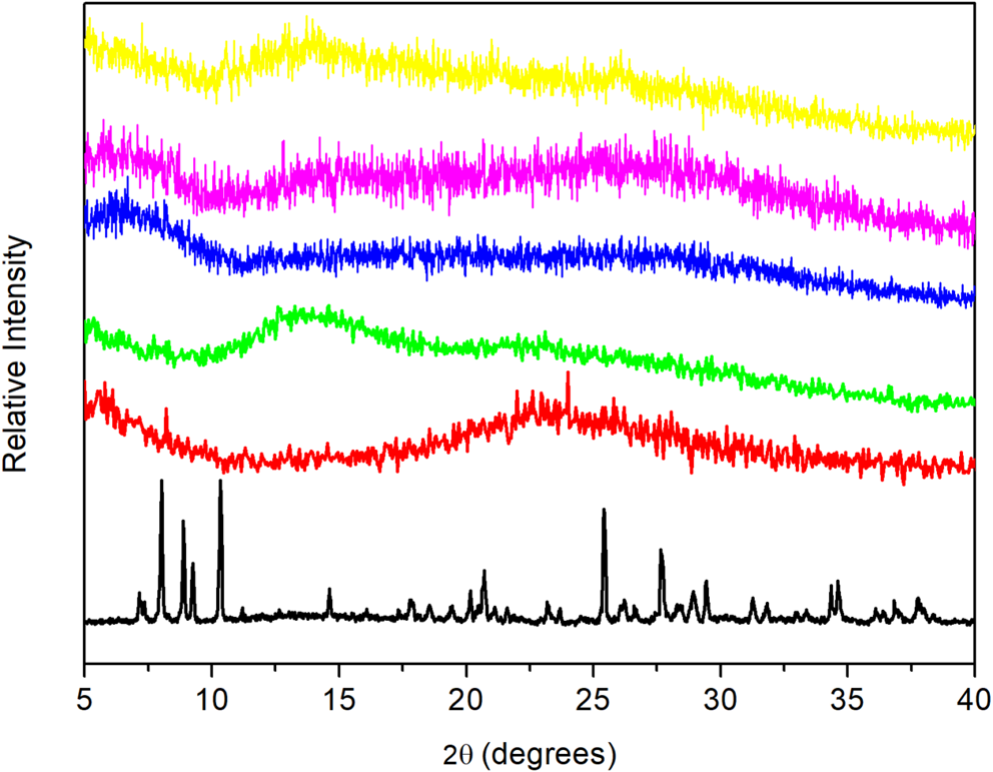
XRD of pure HPW (black), pure silica (red) and 20%HPW@SiO_2_-alcohol catalysts: MeOH (green); EtOH (blue); PrOH (violet); BuOH (yellow).

Powder XRD of *x*%HPW@SiO_2_-alcohol catalysts were very similar, and an example is shown in Figure S3, which used ethanol in the synthesis. Silica displayed only a broad band in the range 20° < 2θ < 30° (centered at 22°). Pure HPW treated at 300 °C for 4 h displayed a well-defined crystalline structure pattern that is probably related to the crystalline phase with more than 6 molecules of water, as known in the literature.^59,62^ The most intense reflections are located at 2θ = 8.2, 10.4, 25.4, and 34.7°. The XRD of 20%HPW@SiO_2_-alcohol catalysts are shown in Figure 2. The pattern of these materials resembled that of pure silica, except for a broad reflection at 5° < 2θ < 10°, not well-defined in our diffractograms. This reflection was explained in the literature as the X-ray scattering by nonordered hydrated polyanions of HPW.^63^ There are either isolated molecular moieties or small clusters with few Keggin units. Crystals of HPW entrapped on the silica surface may be deposited as separate entities, but not big enough or well-formed to be detected by XRD. Hence, HPW incorporated on silica apparently showed structures quite dispersed in the matrix, reducing the size of the HPW crystals. No attempt was made to calculate the size of the crystalline domains by the Scherrer equation, but it is certainly below 5 nm, as observed for HPW supported on a silica–alumina matrix.^22^ Thus, using XRD it was not possible to confirm the presence of HPW in the material, which indicated that another technique should be used to prove it.

#### 3.2.3 Solid State NMR (^31^P MAS NMR)

**Figure 3:**
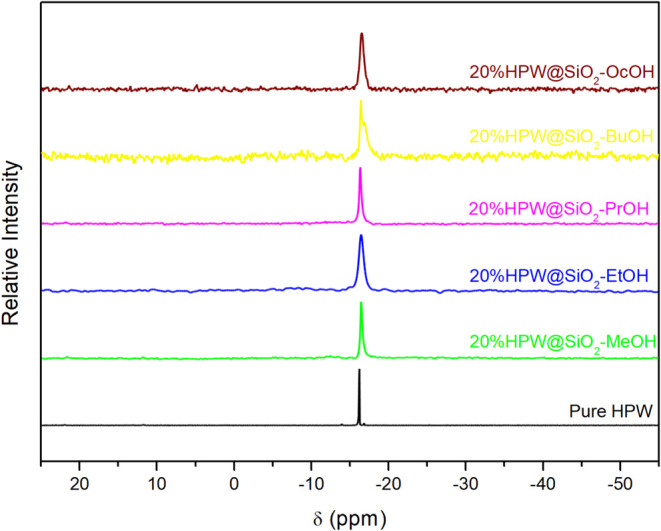
^31^P MAS NMR spectra of hydrated HPW (black) and 20%HPW@SiO_2_-alcohol catalysts: MeOH (green); EtOH (blue); PrOH (violet); BuOH (yellow); OcOH (brown).

^31^P MAS NMR spectroscopy can determine the presence of the Keggin structure for HPW supported on silica and other matrices, better than FT-IR or XRD. The spectra for *x*%HPW@SiO_2_-alcohol catalysts were quite similar, and an example for the preparation using 1-butanol is shown in Figure S4. In HPW n·H_2_O the isotropic shift (δ) value is around −15 ppm, depending on the degree of hydration.^59,64^ The interaction of HPW with SiO_2_ produced a characteristic broad signal at −15.1 ppm in all 20%HPW@SiO_2_-alcohol catalysts (Figure 3), which was attributed to the crystalline environment of ^31^P that is like hydrated HPW.^22^ A single peak of hydrated HPW highlights the mobility of the proton’s environment around phosphorus, since the mobility of the proton through water molecules distributes the electron density in a similar manner around the Keggin anion during the time of the NMR experiment.^17,64^ Hence, the ^31^P MAS NMR spectra unequivocally shows the presence of the Keggin anion in the encapsulated HPW catalysts, as already observed for the supported ones.^59^

### 3.3 Textural Analysis (N_2_ Physisorption at −196 °C)

Textural properties are important features that affect the reactivity in catalytic materials and depend on the material preparation conditions.^65^ These properties of the synthesized 20%HPW@SiO_2_-alcohol catalysts, as well as 20%HPW/SiO_2_ were obtained by low-temperature nitrogen sorption isotherms (Figure S5). The profile of pure HPW was described as combined isotherms of type II and IV(a), which is consistent with macroporous or nonporous solids with low BET area (about 6 m^2^ g^–1^).^60^ The silica gel Aerosil showed BET area of 250 m^2^ g^–1^ with N_2_ sorption curves corresponding to predominant type VI(a) (mesoporous) with type I(b) (microporous) component.

**Figure 4:**
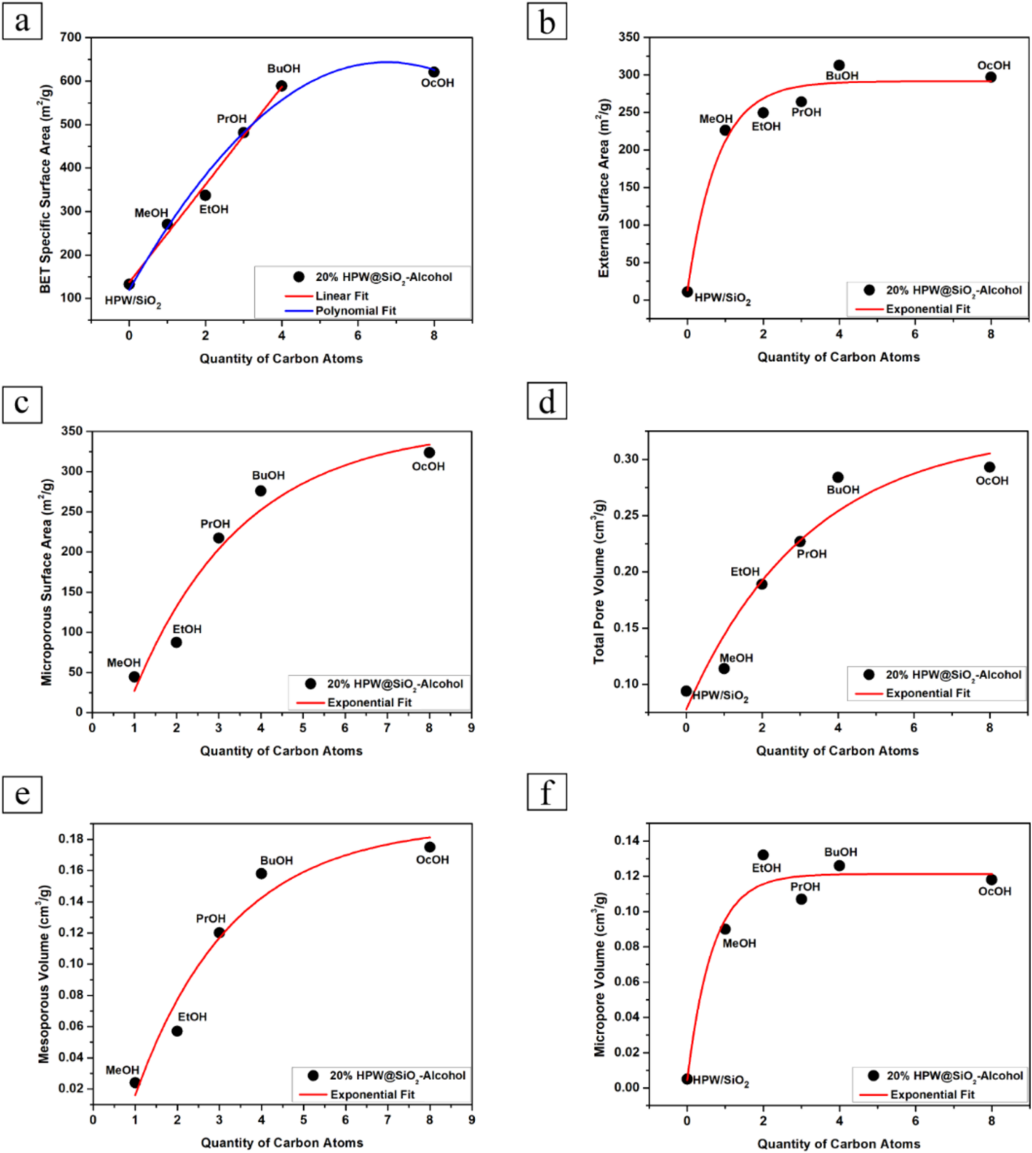
Correlations between textural properties and carbon chain length of alcohols used in the synthesis of 20%HPW@SiO_2_-alcohol: (a) BET specific surface area; (b) external surface area; (c) microporous surface area; (d) total pore volume; (e) mesoporous volume; (f) micropore volume.

The textural data of the composites are compiled in Table 3. First, 20%HPW/SiO_2_ had a sorption curve that was predominantly of a mesoporous solid, whereas the 20%HPW@SiO_2_-alcohol showed a larger amount of micropores (Table 3). The quantity of mesopores increased with the increase in the carbon chain in the synthesis, as observed by the *V*_Meso_ calculation. As a result, the isotherms of the encapsulated HPW had a typical combination of type I(b) with type VI(a) according to IUPAC classifications.^65^ Correlations could be obtained with the textural data (Figure 4). An interesting one was between the BET area versus the carbon chain of the alcohol used in the synthesis (Figure 4a). Clearly there was an increase in the BET area with the number of carbon atoms. In addition, the behavior within 0 to 4 carbons (*n*-butanol) was linear (*r*^2^ = 0.998). This shows that the hydrolysis and further calcination of the composites at 300 °C gave rise to a regular growth of the porous system that contributed to the total specific surface area of these catalysts. A gradual increase in the external area, microporous area and volume, total volume of pores, and mesoporous volume in relation to the quantity of carbon atoms in the alcohol used in the synthesis was also observed (Figure 4b–f) with a tendency toward saturation of these values after 4 carbon atoms. These trends influenced the catalytic activity of the materials in the dehydration of 1-propanol.

**Table 3:** Textural Properties from N_2_ Adsorption/Desorption Isotherms at −196 °C for the Catalysts Calcined at 300 °C

catalyst	*C*^a^ (#)	BET^b^ (m^2^/g)	*S*_micro_^c^ (m^2^/g)	*S*_Ext_^d^ (m^2^/g)	*V*_p_^e^ (cm^3^/g)	*V*_micro_^f^ (cm^3^/g)	*V*_meso_^g^ (cm^3^/g)
20%HPW/SiO_2_	0	132.5	10.9	121.6	0.09	0.01	0.09
20%HPW@SiO_2_-MeOH	1	270.6	226.2	44.4	0.11	0.09	0.02
20%HPW@SiO_2_-EtOH	2	337.1	249.6	87.5	0.19	0.13	0.06
20%HPW@SiO_2_–PrOH	3	481.6	264.2	217.4	0.23	0.12	0.12
20%HPW@SiO_2_–BuOH	4	588.9	312.9	276.0	0.28	0.13	0.16
20%HPW@SiO_2_–OcOH	8	620.6	296.9	323.7	0.29	0.12	0.17

a Quantity of carbon atoms in the alcohol used in the synthesis.

b Specific surface area obtained by the BET method in the *P*/*P*_0_ range of 0.01 to 0.1. The standard error (3σ) was ± 5 m^2^/g.

c Microporous surface area obtained by the *t*-plot method.

d External surface area obtained by the *t*-plot method.

e Total pore volume (*V*_p_) calculated by the quantity of gas adsorbed at *P*/*P*_0_ = 0.98.

f Micropore volume calculated by t-plot method.

g Mesoporous volume calculated by *V*_p_ – *V*_micro_.

The incorporation of HPW clusters during silica gel formation can significantly alter the resulting supramolecular assemblies, leading to more disordered hybrid solids. These supramolecular arrangements involve water and alcohol molecules. Micropores in the material are formed from the porous structure of silica particle gels, which are obtained through the hydrolysis of TEOS under acidic conditions.^26^ The entrapment of polyanions within the silica network reduces pore size within the micropores. The embedded Keggin anions effectively form a highly concentrated aqueous solution within the silica network, narrow enough to prevent the leaching of HPW.^26,27^ The presence of alcohol during synthesis may enhance accessibility to the Keggin anion depending on its chain length, although this effect likely has a limit, as observed in other synthesis processes (e.g., zeolites, OMS). This limitation influences the textural properties of the materials. A more detailed discussion on this topic is provided in Section 3.5 (Effect of Alcohol Chain Length on Sol–Gel Silica Condensation).

### 3.4 Scanning Electron Microscopy (SEM)

**Figure 5:**
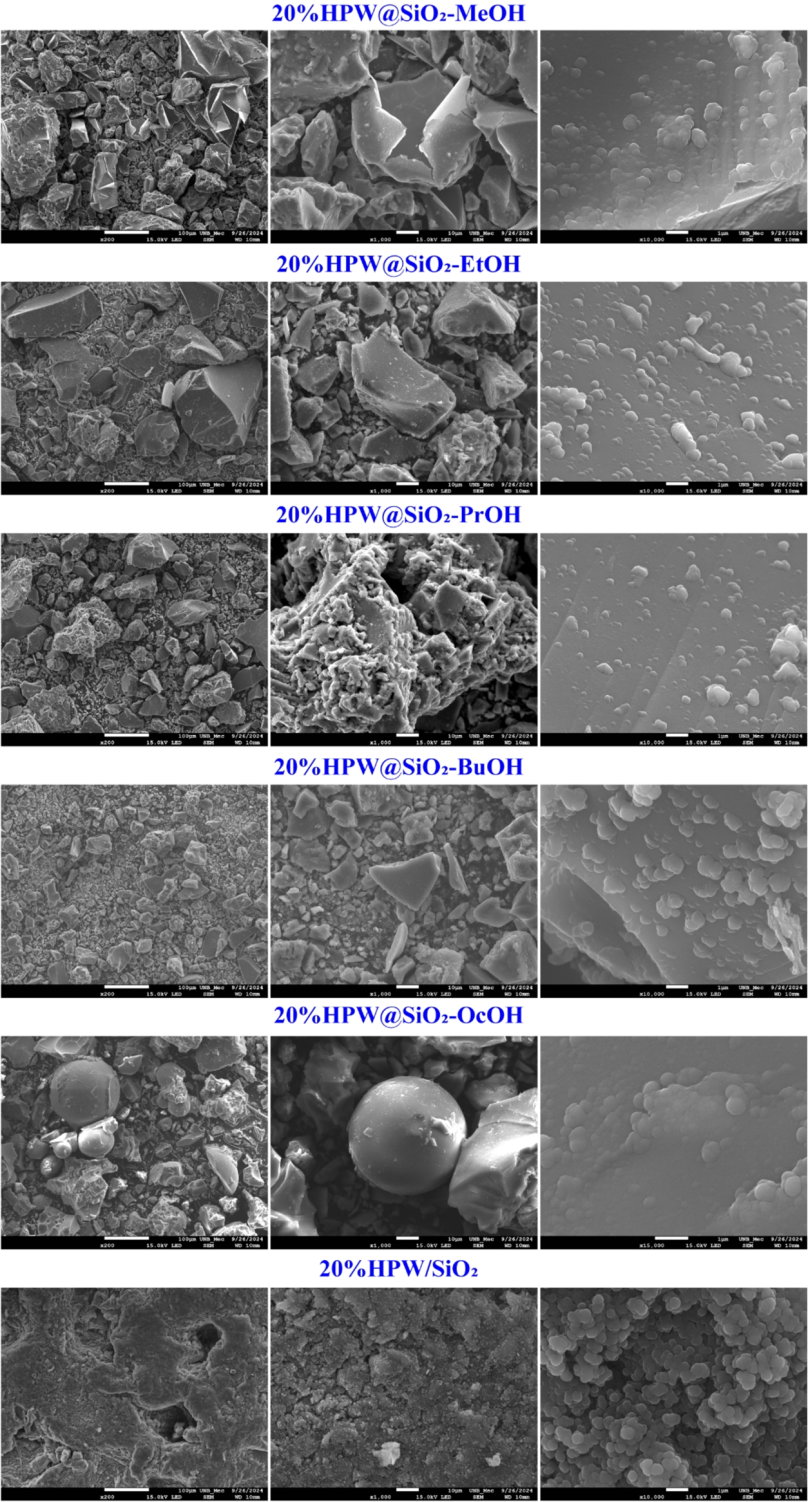
SEM images of 20%HPW@SiO_2_-alcohol under different magnifications.

The SEM images of 20%HPW@SiO_2_-alcohol catalysts under sequential amplifications on a microscale level are shown in Figure 5. At lower magnification, the composites are not very regular in size and shape, but at higher magnification, the grains tend to be approximately spherical with sizes around 0.5 μm and a significant number of agglomerates. It can also be noted that as the syntheses used larger chain alcohols, the particles became more regular in form and size. The morphology of pure silica is also marked by the formation of round to spherical particles, as described in the literature.^66^

**Figure 6:**
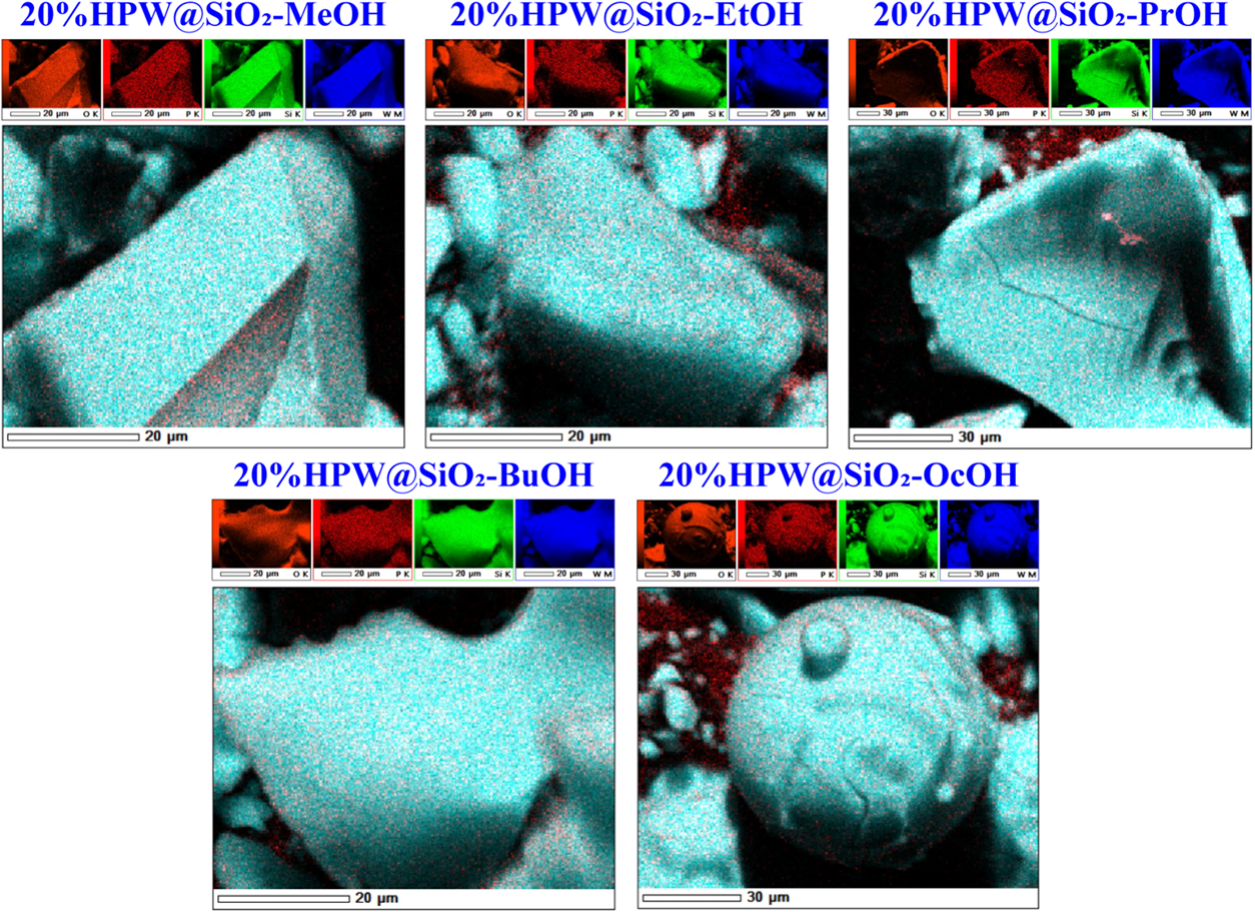
SEM-EDS images of 20%HPW@SiO_2_-alcohol catalysts.

At the magnification used, crystalline HPW could not be detected in the composites, which was similar to observations by Popa et al. in HPA containing Mo and V on the silica surface.^38^ Using X-ray energy dispersive (EDS) in particle domains (20 × 20 μm^2^ or 30 × 30 μm^2^), a highly homogeneous distribution of the elements Si, O, P, and W was observed (Figure 6), reasonably close to the stoichiometric values (based on the EDXRF as the reference for total HPW loading calculation). Deviations may be attributed to heterogeneity due to agglomeration of HPW at some points on the silica surface, since the Keggin–Keggin interaction is very strong compared to Keggin-SiO_2_, as known in the literature.^67^ Nonetheless, these results confirmed the XRD pattern obtained by these composites, i.e., no crystalline HPW phase detected, which may be assigned to the high dispersion of HPW on silica. For comparison, the incipient supported HPW on silica (20%HPW/SiO_2_) showed more round agglomerated particles than the 20%HPW@SiO_2_-alcohol, which is also consistent with previous XRD results that detected some of the typical peaks of the HPW structure in the former.^59^

### 3.5 Effect of Alcohol Chain Length on Sol–Gel Silica Condensation

The hydrolytic polycondensation processes of silica precursors such as tetramethyl orthosilicate (TMOS) or tetraethyl orthosilicate (TEOS) are well-known and documented in the literature.^68−71^ The polymerization of silica precursors such as silicon alkoxides in aqueous and/or alcoholic solutions gives rise to highly cross-linked solids, as described by Dufaud and Lefebvre.^26^ In that article, the authors also reviewed in detail the processes of encapsulated HPA in oxide matrices. Our process involved the formation of nonstructured silica material that took place in an alcoholic solution, but with some water content. The condensation of Si(OR)_4_ alkoxide requires an acid or basic pH. The addition of HPW to the alkoxide solution provided this acidic medium for polymerization. As the HPW dissociates in solution with polar solvents such as the alcoholic medium, the Keggin anion is entrapped into the silica matrix at the same time the chain polymerization grows. There is a strong interaction between the SiO_2_ framework and the Keggin anion, which stabilizes species such as [(≡Si–OH_2_^+^)(H_2_PW_12_O_40_^–^)].^72^ At the same time, the solvent volume is kept approximately constant because an increase in the solvent content increases the spacing between the reacting species. Thus, the process leads to a decrease in the reaction rate and hinders the formation of the gel framework causing longer gelation times.^70^ Therefore, what has been modified in our synthesis is the type of alcohol that led to the formation of gels with different distributions of pores (after calcination), as we have depicted in the textural properties of the materials. As described in the literature, generally these materials present porous in the micropores region (<2 nm), and some diffusional problems may be anticipated.^26,27^ In this study, it was displayed that even in a simple procedure, it is possible to tune the micropores and mesoporous by changing the alcohol during the gel formation. The size of the alcohol chain probably produces a larger spacing around the Keggin anion along the formation of the silica chains and the alcohol and water molecules present in the reaction medium. After drying and calcining the gel, voids (pores) with varying sizes and distributions are formed. As can be seen, the larger the alcohol chain, the greater the tendency to form mesopores. This is analogous to the formation of ordered mesoporous silica (OMS), where the size of the pores can be controlled as a function of the type of surfactant used.^73−75^ A conclusive explanation for the observed saturation of the porous system and its underlying mechanism requires more detailed experiments involving variable quantities of alcohol, water, and HPW. These aspects will be explored in future studies.

### 3.6 Dehydration of 1-Propanol Reaction

Propene is one of the main products produced by petrochemical processes with target applications in polypropylene, acrylonitrile, and acrylic acid, among other chemicals.^55^ Moreover, dehydration of 1-propanol can be a route to produce propene and is applied as a test reaction to probe acidity of solid acids.^58,76^ Thus, this reaction was used to probe acidity of the 20%HPW@SiO_2_-alcohol catalysts.

**Figure 7:**
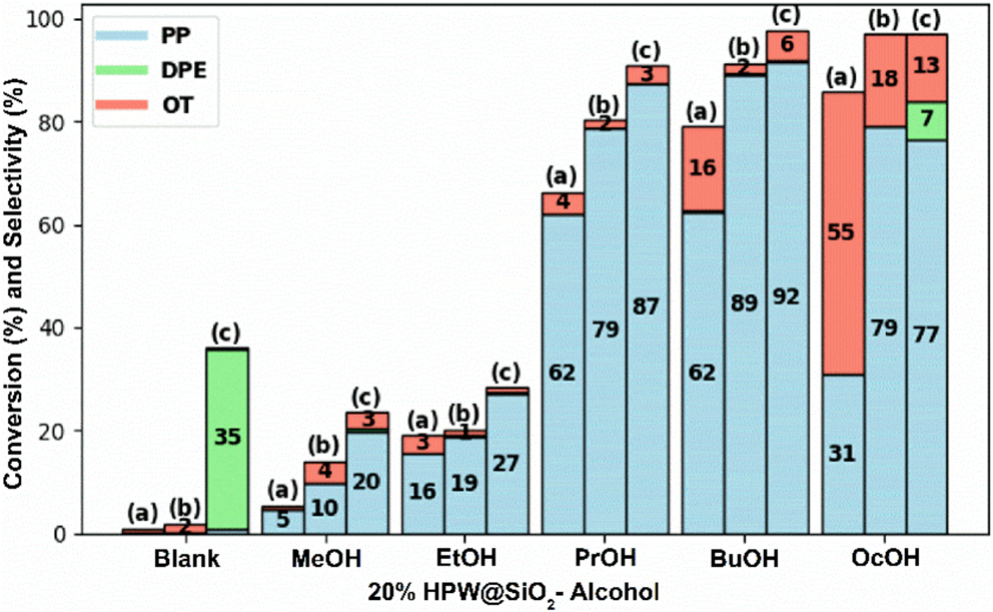
Conversion of 1-propanol and selectivity for all catalysts containing 20%HPW@SiO2-alcohol for propene (PP), dipropyl ether (DPE), and other products (OT) at temperatures of: (a) 300 °C; (b) 350 °C; (c) 400 °C.

The 20%HPW@SiO_2_-alcohol catalysts were used in the 1-propanol dehydration under different temperatures, and the results are organized in Figure 7 and Table S1.

The catalytic reaction was tested at temperatures of 200 and 250 °C, but the conversion was very low and not significant compared to the blank test, under our experimental conditions. It can be noted that the thermal reaction to convert 1-propanol without any catalyst (blank) was negligible at temperatures below 400 °C. At that temperature, the conversion of 1-propanol (about 35%) generated mainly dipropyl ether (DPE) and very little propene (PP). Using the 20%HPW@SiO_2_-alcohol catalysts, the conversion of 1-propanol generally increased as the alcohol chain grew (i.e., from MeOH to OcOH), and this trend was followed by the increasing temperature. Nevertheless, it was observed that the catalyst 20%HPW@SiO_2_–BuOH showed the maximum selectivity to PP (i.e., 62, 89, and 92% at temperatures of 300, 350, and 400 °C, respectively). Additionally, one may look at the performance of the 20%HPW/SiO_2_ catalyst and see that the selectivity to PP at 300, 350, and 400 °C was 24, 65, and 87%, together with generally lower conversions. Moreover, the 20%HPW@SiO_2_–BuOH catalyst had higher conversion of 1-propanol than the 20%HPW/SiO_2_ catalyst in all cases, which indicated that it was the best in the series (highest conversion of 1-propanol, and selectivity to PP).

To check for acid sites in the 20%HPW@SiO_2_-alcohol catalysts, pyridine was adsorbed as per a published procedure,^22^ and FT-IR spectra were obtained (Figure S6). The results indicate that the acid sites are primarily Brønsted, shown by the bands around 1540 and 1488 cm^–1^, consistent with the presence of pyridinium ion, like other supported HPW catalysts.^22,59^ Thus, all catalysts contain potentially active Brønsted acid sites.

**Figure 8:**
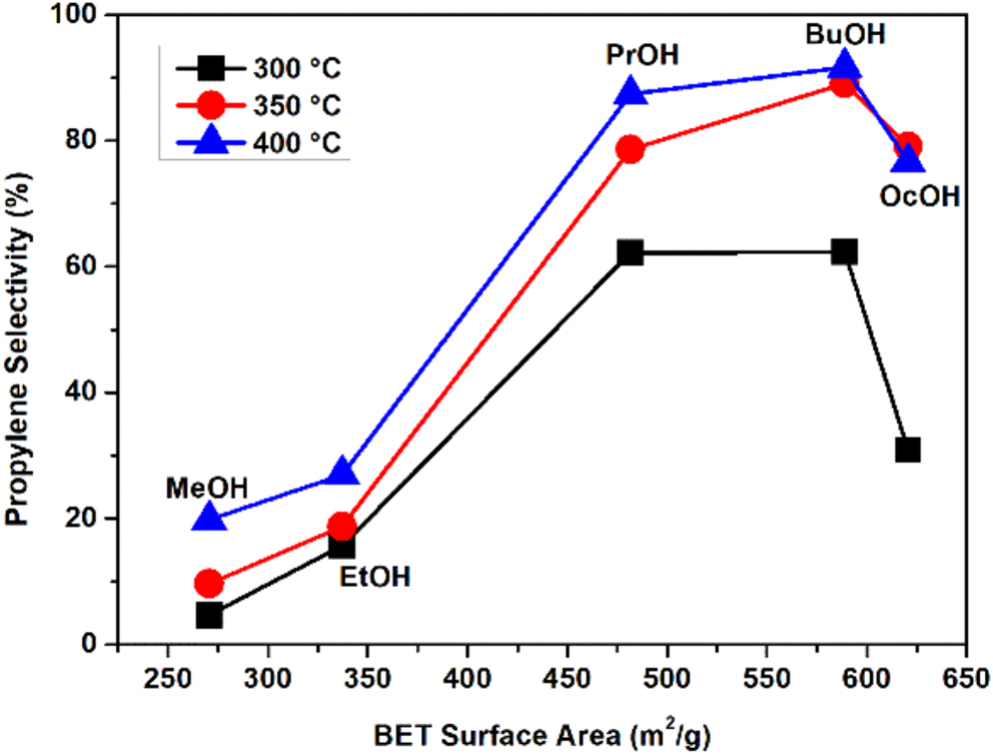
Correlation between the catalytic selectivity for propylene and the BET specific surface area, considering the alcohol chain used in the preparation.

**Figure 9:**
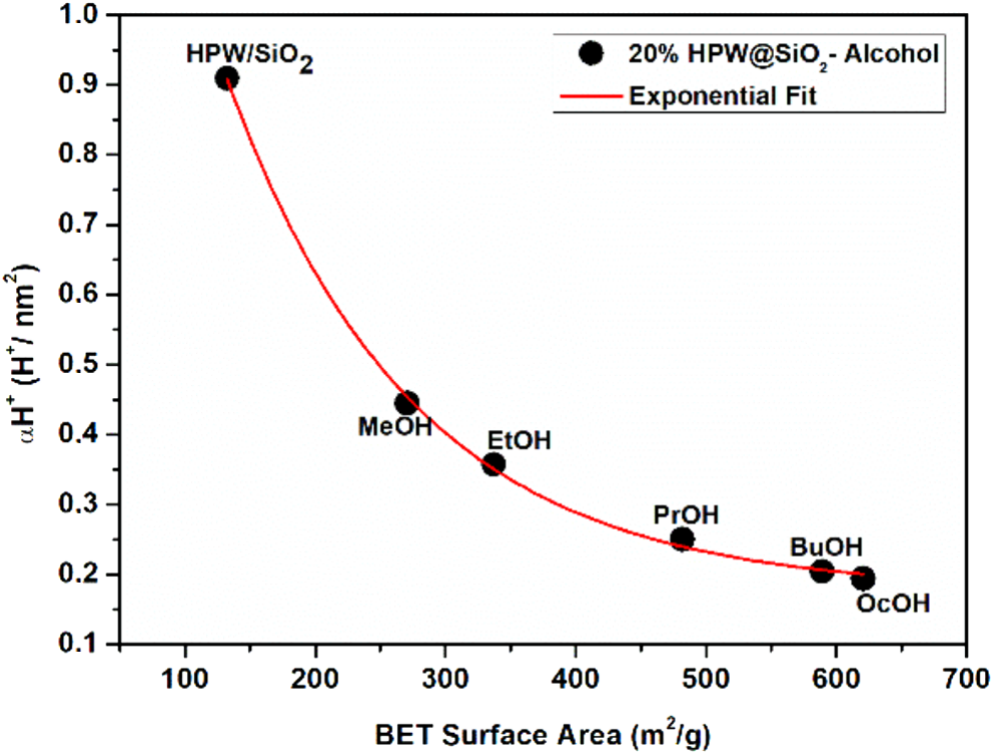
Correlation between the density of Brønsted acid sites (*n*_H^+^_/nm^2^) of the 20%HPW@SiO_2_-alcohol catalysts and their BET specific surface area, considering the alcohol chain used in the preparation.

Considering that all encapsulated and incipient HPW catalysts had about the same number of protons (i.e., Brønsted sites) originated from the nominal 20 wt % loading (i.e., 0.20 mmol g^–1^), the conversion is dependent on the accessibility of these sites. The higher the specific surface area, the easier that accessibility is. The improvement in the conversion may be explained by the increase in some of the textural properties (e.g., specific surface area, total pore volume, mesoporous volume), which tended to reach a maximum when correlated to the number of carbon atoms in the alcohol used in the synthesis (Figure 8). The conversion and selectivity to one product obtained from an acid catalytic reaction is also dependent on the strength and distribution of the acid sites. To find this dependence, the density of acid sites (i.e., the quantity of Brønsted sites divided by the specific surface) was calculated and plotted against the specific surface area of the catalysts (Figure 9). The detailed calculation is provided in the Supporting Information, based on the literature.^75^

Hence, it was possible to observe the same trend forming a plateau region when the preparation used BuOH. In this scenario, there was an optimum value of acid site distribution to convert 1-propanol to PP selectively, which could be estimated by our curve to be about 0.20 H^+^ nm^–2^.

**Figure 10:**
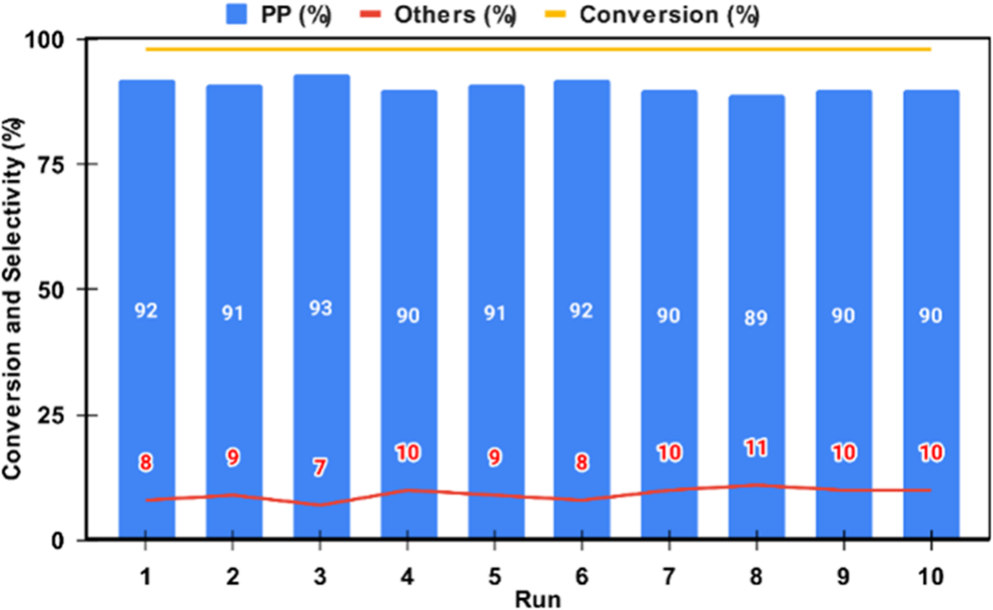
Reutilization of 20%HPW@SiO_2_–BuOH catalyst in ten consecutive 1-propanol dehydration reactions at 400 °C.

The preliminary stability of the 20%HPW@SiO_2_–BuOH catalyst for reutilization was evaluated through ten consecutive injections of 1-propanol at 400 °C (Figure 10). The conversion remained consistent at approximately 97–99%, while the selectivity for propylene was maintained between 89–93%. Although the catalyst demonstrated stable performance, no structural analysis has been conducted to date.

A brief comparison of our results with other catalysts used in the same reaction, as reported in the literature,^77−80^ is provided in Table 4. Interestingly, there are relatively few recent articles focusing on this reaction, with a significantly larger number of studies dedicated to isopropanol dehydration. It should be noted that the reaction conditions are not always identical due to differences in reactor operational design and thermodynamic parameters (e.g., temperature, pressure). The data highlight the potential of our 20%HPW@SiO_2_–BuOH catalyst for 1-propanol dehydration, achieving an average conversion of 98 and 91% selectivity for propene. The primary advantage of this catalyst lies in its high selectivity for propene and efficient 1-propanol conversion, while the main drawback is the relatively high operating temperature.

**Table 4:** Conversion (*C*) and Selectivity(Sel) to Propene from Some Catalysts That Were Used in the Gas-Phase 1-Propanol Dehydration

catalyst	*C* (%)	Sel (%)	*T* (°C)	comments	references
20%HPW@SiO_2_–BuOH	98	92	400	a	this work
HPW	70	43	170	b	(57)
AM-11	63	100	200	c	(77)
Al-MCM-41	100	100	400	d	(78)
M-HZSM-5	100	97	230	e	(79)
HSO_3_/SiO_2_	39	66	100	f	(80)

a Catalyst calcined at 300 °C; WHSV = 0.7 h^–1^; *P* = 95.6 kPa.

b Catalyst calcined at 200 °C for 2 h; *P* = 101 kPa.

c Niobium silicate catalyst activated at 400 °C; WHSV = 0.4 h^–1^; TOS = 60 min.

d Catalysts with different Si/Al ratios (e.g., 237); GHSV = 16,400 h^–1^.

e Catalysts M-HZSM-5 (M = H, V, Cu, or Zn), Si/Al = 12.

f SiO_2_ functionalized with sulfonic acid groups calcined at 550 °C.

Other studies have focused on the catalytic mechanism of 1-propanol dehydration, highlighting the preferential formation of products, which depends on the catalyst composition.^81−85^ Although various factors such as thermodynamic conditions, theoretical studies, and catalyst types are considered in detail, acidity generally plays a crucial role in the reaction, with Brønsted sites being key to the transformation of 1-propanol into olefins or ether products. The stability of the carbocation-like transition state is the primary factor influencing the rates of unimolecular dehydration, leading to enhanced selectivity for olefin formation. The reaction begins with the adsorption of 1-propanol as an alkoxy species, followed by water elimination (E1) and subsequent desorption of propene. Additional routes, including the formation of dimers or even trimers of adsorbed 1-propanol, may also be present, depending on the pressure used in the experimental setup. The strength of the acid sites and the pore structure of the catalyst are essential in defining the exact mechanism of this reaction on solid acids.

## 4 Conclusions

In this study, we investigated the incorporation of 12-tungstophosphoric acid (HPW) into a silica matrix using different alcohols (methanol, ethanol, 1-propanol, 1-butanol, and 1-octanol) at various loadings (10 to 40 wt %). Characterization by several methods confirmed that the Keggin structure of HPW was preserved in all preparations. SEM images of the 20%HPW@SiO_2_-alcohol catalysts revealed approximately spherical particles (∼0.5 μm) with good dispersion in the composites. Elemental and leaching tests detected 20%HPW@SiO_2_–BuOH as one of the most promising materials for catalysis. The textural study showed that pore size and volume, particularly mesoporous characteristics, increased with the carbon chain length of the alcohol used during synthesis. A linear correlation of BET specific surface area with the number of carbon atoms of the alcohol used in the synthesis was observed up to four carbons, with saturation trends in other parameters occurring beyond this point. This effect is attributed to the alcohol chain size, which creates voids (pores) during gel formation and further calcination, enhancing mesopore formation. These mesopores likely improve the accessibility of 1-propanol to Brønsted acid sites, which contributes to the superior performance of 20%HPW@SiO_2_–BuOH. At 400 °C, this catalyst achieved a 1-propanol conversion of ∼98% with 92% selectivity for propene, having a Brønsted acid site density of ∼0.20 H^+^ nm^–2^. The combination of specific surface area, acid density, and dispersion appears to be the optimum parameters for the catalytic reaction under our experimental conditions. In a series of ten consecutive pulsed dehydration reactions, the catalyst maintained consistent conversion (97–99%) and selectivity to propene (89–93%). Future studies for these composites should include: (i) structural analysis of the spent catalyst; (ii) comprehensive acidity studies for the *x*%HPW@SiO_2_–BuOH series (*x* = 10, 20, 30, and 40 wt %); (iii) increased pulsed tests in the microreactor to evaluate long-term stability.

## Supporting Information Available

Analytical equations used for calculating leaching experiments; FT-IR spectra of *x*%HPW@SiO_2_–BuOH with (a) 40%, (b) 30%, (c) 20%, and (d) 10% HPW loading (Figure S1); comparative FT-IR spectra of 20%HPW@SiO_2_–OcOH with 20%HPW/SiO_2_ (Figure S2); XRD patterns of x%HPW@SiO_2_-EtOH of: (a) 10%, (b) 20%, (c) 30%, and (d) 40% HPW loading (Figure S3); ^31^P MAS NMR spectra *x*%HPW@SiO_2_–BuOH with 10, 30%, and 40% HPW loading (Figure S4); isotherms of nitrogen adsorption/desorption (−196 °C) of 20%HPW@SiO_2_-alcohol (Figure S5); FT-IR spectra after pyridine adsorption of 20%HPW@SiO_2_-alcohol and 20%HPW/SiO_2_ (Figure S6); conversion of 1-propanol and selectivity to propene (PP) and dipropyl ether (DPE) for dehydration at different temperatures using 20%HPW@SiO_2_-alcohol catalysts (Table S1); equation for calculation of the density (α_H^+^_) of Brønsted acid sites (*n*_H^+^_/nm^2^) (PDF)
