# Supplementary material for: Preparation of 12-Tungstophosphoric Acid Embedded in a Silica Matrix and Its Effect on the Activity of 1-Propanol Dehydration
Source: ACS Omega. 2025 Apr 14;10(16):16277–90. doi: 10.1021/acsomega.4c10379 (PMC12044439; doi:10.1021/acsomega.4c10379)
Supplement: Supplementary file 1 — ao4c10379_si_001.pdf [file ao4c10379_si_001.pdf]

Preparation of 12-tungstofosforic acid embedded in a silica matrix and its effect on the activity of 1-propanol dehydration

Eduardo de Souza Mello Falcão [a], Deborah da Silva Valadares [a], Giovana Magalhães dos Santos [a], Estelle Silva Diorato Teixeira de Mendonça [b], Marcello Moreira Santos [c], Sílvia Cláudia Loureiro Dias [a*] and, José Alves Dias [a*]

Addresses:

[a] Universidade de Brasília, Campus Universitário Darcy Ribeiro, Asa Norte, Instituto de Química, Laboratório de Catálise, Brasília-DF, 70910-900, Brazil.

* E-mails: jdias@unb.br or josediasunb@gmail.com;
scdias@unb.br or silviadiasunb@gmail.com

[b] Universidade Federal da Bahia, Instituto de Química, Rua Barão de Jeremoabo, 147, Campus Universitário de Ondina, Salvador-BA, 40170-115, Brazil.

[c] Universidade de Brasília – Campus Universitário Darcy Ribeiro – Asa Norte - Instituto de Química - Laboratório de Degradação e Estabilização de Compostos; Brasília-DF, 70910-900, Brazil.

Supporting Information

EXPERIMENTAL

Leaching of the catalysts

The analytical equations obtained for each solution of HPW in the respective solvent, and the correlation coefficient were:

Methanol: Absorbance = 5.03 x10^4 [HPW] – 0.0412 (r^2 = 0.993)

Ethanol: Absorbance = 4.27 x10^4 [HPW] + 0.2542 (r^2 = 0.996)

1-Propanol: Absorbance = 4.63 x10^4 [HPW] + 0.0375 (r^2 = 0.997)

1-Butanol: Absorbance = 4.17 x10^4 [HPW] + 0.0254 (r^2 = 0.998)

The calculated limit of detection (3σ) was about 3 ppm of HPW, considering three times the lowest absorbance reading in the curve.

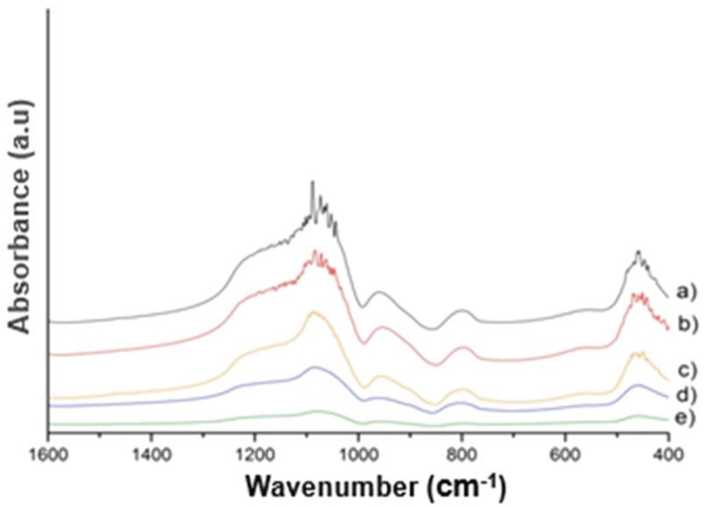

Figure S1. FT-IR spectra of x%HPW@SiO_2-BuOH with: (a) 40%, (b) 30%, (c) 20%, and (d) 10% HPW loading. Pure silica is shown as a reference (e).

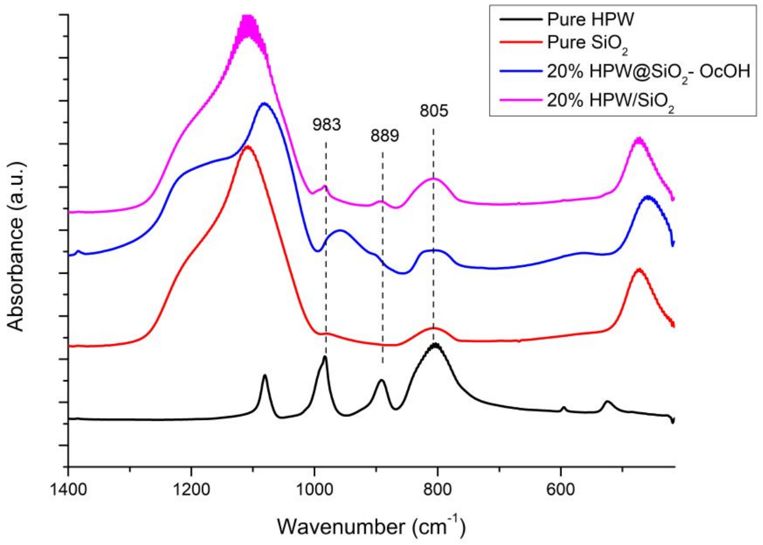

Figure S2. Comparative FT-IR spectra of 20%HPW@SiO_2-OcOH with 20%HPW/SiO_2. Pure SiO_2 and HPW are plotted as references.

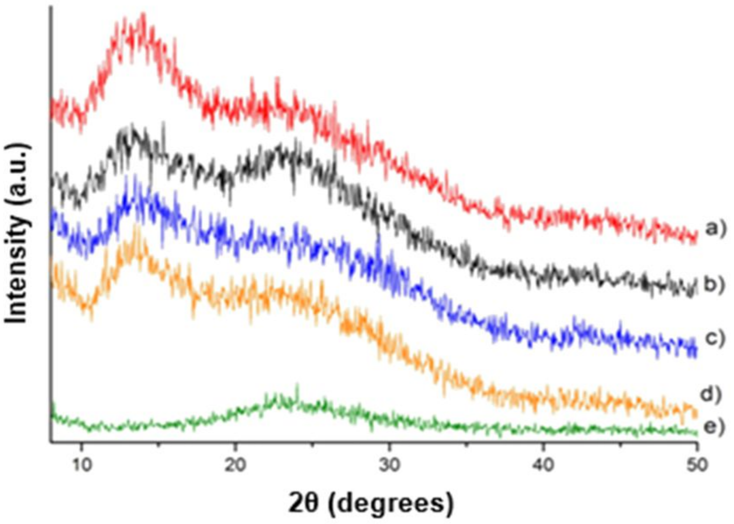

Figure S3. XRD patterns of x%HPW@SiO_2-EtOH of: (a) 10%, (b) 20%, (c) 30%, and (d) 40% HPW loading. Pure silica is shown as a reference (e).

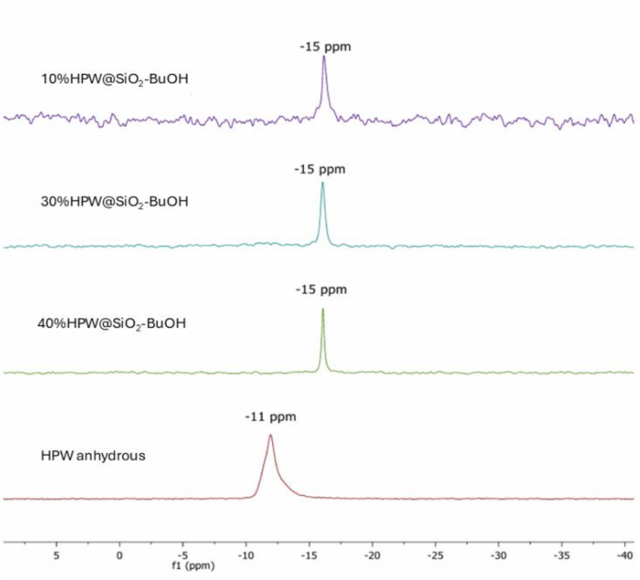

Figure S4. ^{31}P MAS NMR spectra x%HPW@SiO_2-BuOH with 10, 30%, and 40% HPW loading. Note that pure anhydrous HPW shows a signal at -11 ppm.

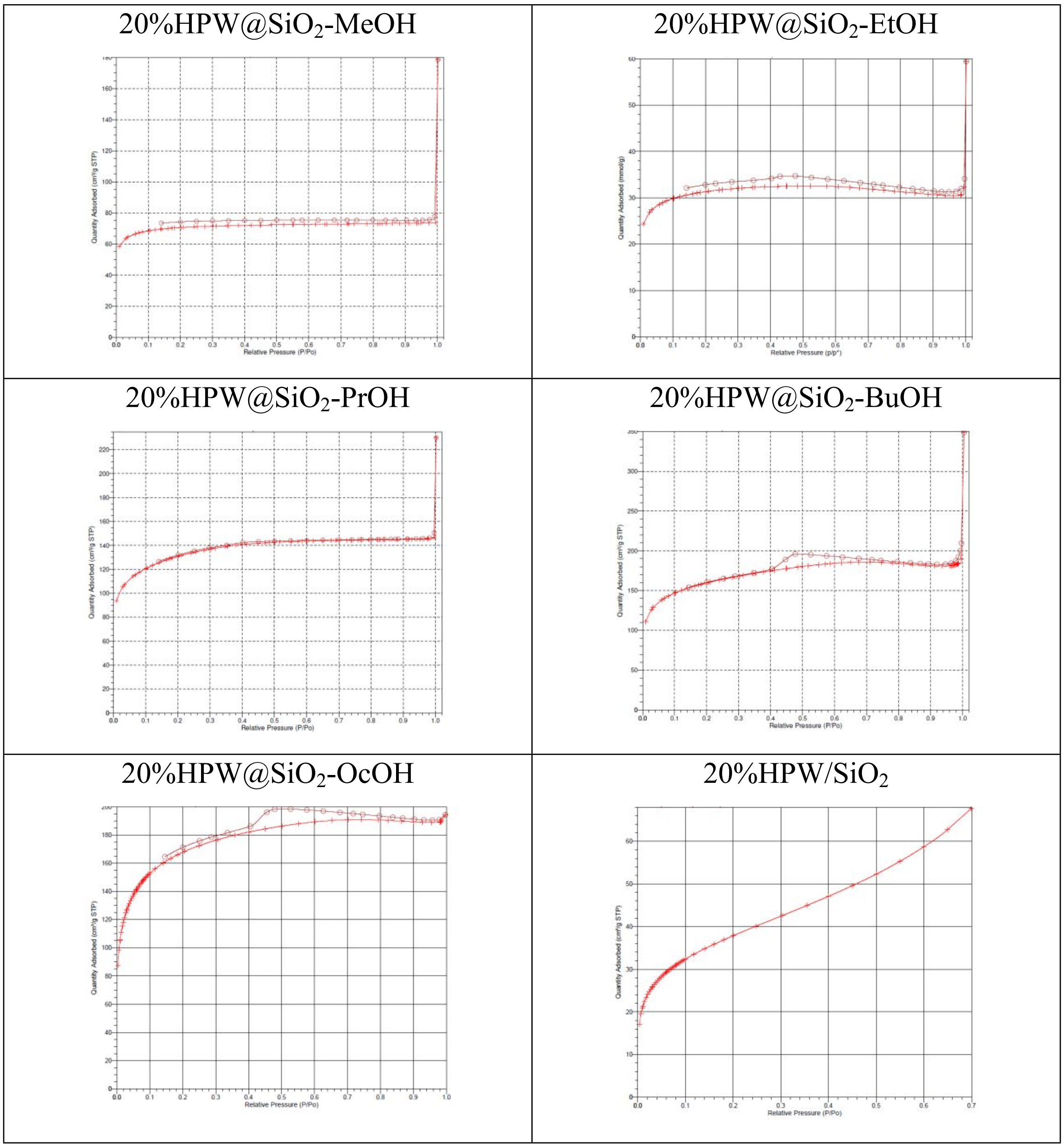

Figure S5. Isotherms of nitrogen adsorption/desorption (-196 °C) of 20%HPW@SiO_2-alcohol and 20%HPW/SiO_2 (prepared by incipient impregnation).

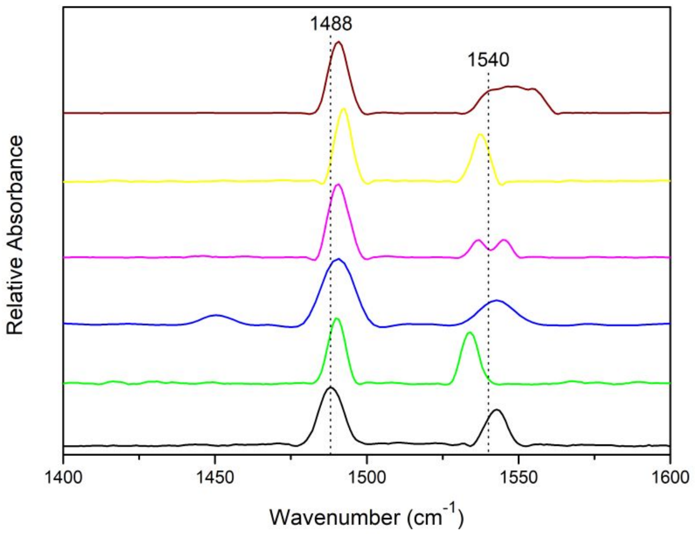

Figure S6. FT-IR spectra after pyridine adsorption of 20%HPW/SiO_2 (black) and 20%HPW@SiO_2-alcohol: MeOH (green); EtOH (blue); PrOH (violet); BuOH (yellow); OcOH (brown).

Table S1 (A-C). Conversion of 1-propanol (C) and selectivity (Sel) to propene (PP) and dipropyl ether (DPE) for dehydration at different temperatures using 20%HPW@SiO_2-alcohol catalysts. Other products (OT) were not determined in our GC analysis.

(A) Temperature = 300 °C

Catalyst	C (%)	Sel PP (%)	Sel DPE (%)	OT (%)
Blank [a]	1.0	0.2	0.0	0.8
20%HPW@SiO_2-MeOH	5.5	4.7	0.1	0.7
20%HPW@SiO_2-EtOH	19.1	15.7	0.0	3.4
20%HPW@SiO_2-PrOH	66.3	62.2	0.0	4.1
20%HPW@SiO_2-BuOH	79.0	62.3	0.3	16.4
20%HPW@SiO_2-OcOH	85.7	30.9	0.0	54.8
20%HPW/SiO_2	47.8	23.9	0.0	23.9

(B) Temperature = 350 °C

Catalyst	C (%)	Sel PP (%)	Sel DPE (%)	OT (%)
Blank [a]	1.9	0.3	0.0	1.6
20%HPW@SiO_2-MeOH	14.0	9.7	0.2	4.1
20%HPW@SiO_2-EtOH	20.1	18.8	0.2	1.1
20%HPW@SiO_2-PrOH	80.3	78.6	0.0	1.7
20%HPW@SiO_2-BuOH	91.4	89.1	0.2	2.1
20%HPW@SiO_2-OcOH	97.0	79.1	0.0	17.9
20%HPW/SiO_2	66.5	65.1	0.4	1.0

(C) Temperature = 400 °C				
**Catalyst**	**C (%)**	**Sel PP (%)**	**Sel DPE (%)**	**OT (%)**
Blank [a]	36.1	0.8	35.1	0.2
20%HPW@SiO_2-MeOH	23.5	19.8	0.5	3.2
20%HPW@SiO_2-EtOH	28.3	27.0	0.4	0.9
20%HPW@SiO_2-PrOH	90.8	87.4	0.1	3.3
20%HPW@SiO_2-BuOH	97.8	91.7	0.3	5.8
20%HPW@SiO_2-OcOH	97.0	76.6	7.4	13.0
20%HPW/SiO_2	89.5	87.4	0.5	1.6

Calculation of the density (α_{H+}) of Brønsted acid sites (n_{H+}/nm^2), based on reference 75:

$$\alpha_{H+} = n_{H+} \times NA \times 10^{-18}/S_{BET}$$

where:

n_{H+} is the number of H^+ sites onto the solid ($10^{-3} \times$ mmol g^{-1})

NA is the Avogadro constant (6.022 x10^{23} mol^{-1})

S_{BET} is the BET specific surface area (m^2 g^{-1})

10^{-18} is a conversion from m^2 to nm^2

n_{H+} = 0.20 mmol/g for 20% HPW@SiO_2-alcohol; anhydrous HPW has 1.04 mmol H^+/g
